# Comparison of diagnostic performance between Oncomine Dx target test and AmoyDx panel for detecting actionable mutations in lung cancer

**DOI:** 10.1038/s41598-024-62857-8

**Published:** 2024-05-30

**Authors:** Yuki Nagakubo, Yosuke Hirotsu, Mona Yoshino, Kenji Amemiya, Ryota Saito, Yumiko Kakizaki, Toshiharu Tsutsui, Yoshihiro Miyashita, Taichiro Goto, Masao Omata

**Affiliations:** 1 Division of Genetics and Clinical Laboratory, Yamanashi Central Hospital, 1-1-1 Fujimi, Kofu, Yamanashi Japan; 2 Genome Analysis Center, Yamanashi Central Hospital, 1-1-1 Fujimi, Kofu, Yamanashi 400-8506 Japan; 3 Lung Cancer and Respiratory Disease Center, Yamanashi Central Hospital, 1-1-1 Fujimi, Kofu, Yamanashi Japan; 4 Department of Gastroenterology, Yamanashi Central Hospital, 1-1-1 Fujimi, Kofu, Yamanashi Japan; 5 The University of Tokyo, 7-3-1 Hongo, Bunkyo-ku, Tokyo, Japan

**Keywords:** Lung cancer, Diagnostic, Oncomine, Amoy, False positive, Diagnosis, Cancer, Diagnostic markers, Lung cancer

## Abstract

Companion diagnostic (CDx) tests play important roles in identifying oncogenic driver genes and tailoring effective molecularly targeted therapies for lung cancer patients. In Japan, the Oncomine Dx target test (ODxTT) and the AmoyDx pan lung cancer PCR panel (AmoyDx) are prominent CDx tests and only one of these tests is covered by the domestic insurance system. However, these CDx tests cover different target regions and apply different technologies (ODxTT is amplicon-based next-generation sequencing and AmoyDx is multiplex PCR-based assay), which may lead to missing of actionable mutations affecting patient prognosis. Here, we performed a direct comparison analysis of 1059 genetic alterations of eight driver genes from 131 samples and evaluated the concordance between two CDx tests for detecting actionable variants and fusions. When excluding the eight uncovered variants (ODxTT: two variants, AmoyDx: six variants), the overall percent agreement was 97.6% (1026/1051) with 89.0% of overall positive percent agreement (89/100) and 98.5% of overall negative percent agreement (937/951). Of the 25 discordant genetic alterations, two were undetected despite being covered in the AmoyDx (one *EGFR* variant and one *ROS1* fusion). Furthermore, there were potential false positives in the ODxTT (nine *MET* exon 14 skippings) and in the AmoyDx (five variants, six *ROS1* and three *RET* fusions). These potential false positives in the AmoyDx likely due to non-specific amplification, which was validated by the unique molecular barcoding sequencing. The ODxTT missed two uncovered *EGFR* rare variants, which was visually confirmed in the raw sequencing data. Our study provides insights into real-world performance of CDx tests for lung cancer and ensures reliability to advance precision medicine.

## Introduction

Lung cancer is the leading cause of cancer-related deaths worldwide, accounting for the highest mortality rates^1^. The two main types of lung cancer are non-small cell lung cancer (NSCLC) and small cell lung cancer, each characterized by distinct genetic features^2,3^. In NSCLC, oncogenic driver mutations are commonly found in genes such as epidermal growth factor receptor (*EGFR*), erb-b2 receptor tyrosine kinase 2 (*HER2*), v-raf murine sarcoma viral oncogene homologue B1 (*BRAF*), Kirsten rat sarcoma viral oncogene homolog (*KRAS*), anaplastic lymphoma kinase (*ALK)*, ROS proto-oncogene 1 (*ROS1*), ret proto-oncogene (*RET)* and hepatocyte growth factor receptor (*MET*)^4^. Identifying the genetic alterations for lung cancer is the first critical step in tailoring effective molecularly targeted therapy to patients^5–13^.

Companion diagnostic (CDx) testing is especially important in lung cancer, because molecular profiling has been shown to benefit patient prognosis^14^. To success the implementation of CDx testing, multiple studies have been conducted on the sample collection, fixation conditions and nucleic acid quality^14–21^. In Japan, two prominent CDx tests, the Oncomine Dx target test (ODxTT) and the AmoyDx Pan Lung Cancer PCR Panel (AmoyDx), are used for molecular profiling in lung cancer^22,23^. These two tests apply different technologies; the ODxTT is based on amplicon-based next-generation sequencing (NGS)^24^, while the AmoyDx is based on amplification refractory mutation system (ARMS) with multiplex real-time PCR^25^. Under the insurance system in Japan, only one of these two CDx tests is covered per patient by national health insurance. The decision of which CDx test to choose is at the physician’s discretion. However, given the differences in the mutations covered by the two CDx tests, physicians are often faced with challenges in their decision-making^26^. To overcome this situation, it is imperative to obtain information on direct performance assessments between these two CDx tests using real-world samples.

In this study, our objective is to conduct a comparative analysis of the ODxTT and the AmoyDx for lung cancer. Specifically, we systematically examine the agreement and disagreements between the ODxTT and the AmoyDx in identifying crucial actionable variants and fusions. These results provide insights that not only contribute to diagnostic practice in current lung cancer care, but also provide valuable perspectives to guide future advances in molecular diagnostics and precision medicine.

## Results

### Evaluation of nucleic acid quantity

To check the quantities of extracted DNA and RNA, we measured the nucleic acids concentrations. The ODxTT and the AmoyDx use different devices for nucleic acid concentration assessment—specifically, Qubit in the ODxTT and NanoDrop in the AmoyDx. The median DNA concentration measured 24 ng/µL (IQR: 17–47 ng/µL) using Qubit and 181 ng/µL (IQR: 120–288 ng/µL) using NanoDrop. For RNA, the median concentration was 42 ng/µL (IQR: 21–93 ng/µL) with Qubit and 97 ng/µL (IQR: 43–167 ng/µL) with NanoDrop. Compared to Qubit, the median concentration value of DNA was 7.5 times higher and that of RNA was 2.3 times higher in NanoDrop. The nucleic acid concentrations measured by Qubit and NanoDrop showed a positive correlation for DNA (Supplementary Fig. 1A, r = 0.68, p < 2.2 × 10^–16^) and RNA (Supplementary Fig. 1B, r = 0.48, p < 6.9 × 10^–9^).

### Performance between ODxTT and AmoyDx

**Figure 1:**
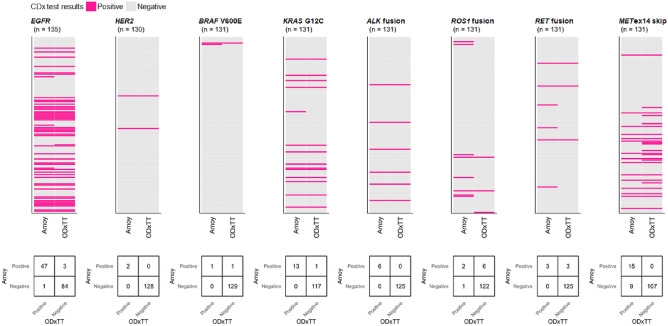
Comparison of test results between ODxTT and AmoyDx. The upper panel shows the results for each gene, indicating positive (pink) or negative (gray) results. The vertical axis represents the tested samples, while the horizontal axis shows the results of the AmoyDx (left column) test and the ODxTT (right column). The cross-table in the lower panel shows the concordance between positive and negative results in two CDx tests. For *EGFR* mutations, if a sample had two mutations, each was evaluated individually. In this figure, mutations that are not covered by either of the CDx tests are excluded.

To examine the concordance between the ODxTT and the AmoyDx, we examined eight target genes (*EGFR*, *HER2*, *BRAF*, *KRAS*, *ALK*, *ROS1*, *RET* and *MET*). When excluding mutations not covered by either test, the number of genetic alterations determined as positive by the ODxTT were *EGFR* (n = 48), *HER2* (n = 2), *BRAF* V600E (n = 1), *KRAS* G12C (n = 13), *ALK* fusion (n = 6), *ROS1* fusion (n = 3), *RET* fusion (n = 3) and *MET*ex14 skipping (n = 24) (Fig. 1). Among these, the concordance with the AmoyDx results was as follows: *EGFR* (positive: 47, negative: 1; positive percent agreement [PPA] = 98% [95% CI 89–100%]), *HER2* (positive: 2; PPA = 100% [16–100%]), *BRAF* V600E (positive: 1; PPA = 100% [2–100%]), *KRAS* G12C (positive: 13; PPA = 100% [75–100%]), *ALK* fusion (positive: 6; PPA = 100% [54–100%]), *ROS1* fusion (positive: 2, negative: 1; PPA = 67% [9–99%]), *RET* fusion (positive: 3; PPA = 100% [29–100%]), and *MET*ex14 skipping (positive: 15, negative: 9; PPA = 63% [41–81%]). The overall percent agreement for positive results between the ODxTT and the AmoyDx was 89% (89 of 100 alterations).

On the other hand, the genetic alterations identified as negative by the ODxTT were *EGFR* (n = 87), *HER2* (n = 128), *BRAF* V600E (n = 130), *KRAS* G12C (n = 118), *ALK* fusion (n = 125), *ROS1* fusion (n = 128), *RET* fusion (n = 128) and *MET*ex14 skipping (n = 107) (Fig. 1). Among these variants, the concordance with AmoyDx results was as follows: *EGFR* (positive: 3, negative: 84; negative percent agreement [NPA] = 97% [95% CI 90–99%]), *HER2* (negative: 128; NPA = 100% [95% CI 97–100%]), *BRAF* V600E (positive: 1, negative: 129; NPA = 99% [95% CI 96–100%]), *KRAS* G12C (positive: 1, negative: 117; NPA = 99% [95% CI 95–100%]), *ALK* fusion (negative: 125; NPA = 100% [95% CI 97–100%]), *ROS1* fusion (positive: 6, negative: 122; NPA = 95% [95% CI 90–98%]), *RET* fusion (positive: 3, negative: 125; NPA = 98% [95% CI 93–100%]), and *MET*ex14 skipping (negative: 107; NPA = 100% [95% CI 97–100%]). The overall percent agreement for negative results between the ODxTT and the AmoyDx was 98.5% (937 of 951 alterations).

### Relationship between variant allele fraction (VAF) and cycle threshold (Ct) values in ODxTT and AmoyDx

To explore the correlation between VAFs from the ODxTT and Ct values from the AmoyDx, we focused on *EGFR* (n = 47) and *KRAS* G12C (n = 13), both included a high number of detected variants than other genes. Ct values decreased along with VAF increased, indicating a higher quantity of mutant allele DNA in sample (Supplementary Fig. 2A and B). The correlation coefficients were – 0.76 (p = 7.1 × 10^–10^) for *EGFR* variants and – 0.82 (p = 5.4 × 10^–4^) for *KRAS* G12C, showing a negative correlation between VAF and Ct values.

### Mutations not covered by the CDx tests

**Figure 2:**
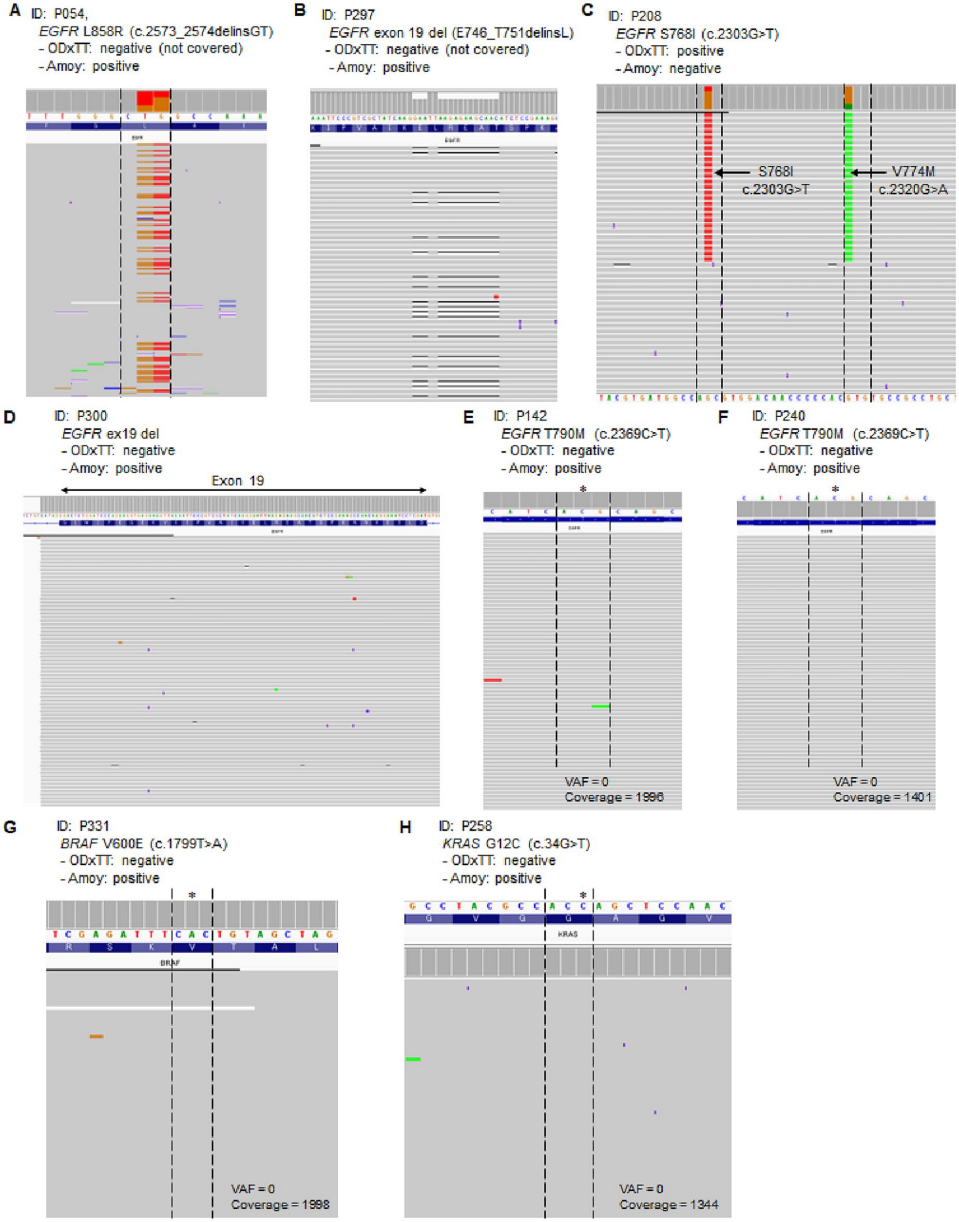
Visual confirmation of discordant results in ODxTT raw sequence data. The sequence read alignments are visualized using integrative genomics viewer (IGV). Eight representative samples are shown. (**A**) *EGFR* L858R mutation was negative by ODxTT and positive by AmoyDx (ID: P054). c.2573_2574delinsGT are shown in orange and red and this dinucleotide change is not covered by ODxTT. (**B**) *EGFR* exon 19 deletion (E746_T751delinsL) was negative by ODxTT and positive by AmoyDx (ID: P297). The black line indicates deletion site, which is not covered by ODxTT. (**C**) *EGFR* S768I mutation was positive by ODxTT and negative by AmoyDx (ID: P208). *EGFR* V774M co-mutation was present in the same allele. Red color shows single nucleotide change (c.2303G > T) and green color shows single nucleotide change (c.2320G > A). (**D**–**H**) Five variants were putative false positives in AmoyDx. Asterisks indicate mutation positions expected to be detected by AmoyDx. The images show *EGFR* exon 19 region (ID: P300) (**D**), *EGFR* T790M (ID: P142 and P240) (**E** and **F**), *BRAF* V600E (ID: P331) (**G**) and *KRAS* G12C (ID: P258) (**H**). The black dashed lines represent the codon corresponding to each mutation. There appear to be no mutant allele reads in the raw data. ID number corresponds to Table 1.

Since the ODxTT and the AmoyDx target different gene mutations for analysis, we examined whether either test failed to detect certain variants. There were six variants covered by the ODxTT but not covered by the AmoyDx. These variants included *EGFR* E709A (c.2126A > C) (n = 2; P094, P320), *EGFR* E709G (c.2126A > G) (n = 2; P251, P269), *EGFR* E709K (c.2125G > A) (n = 1; P279) and *HER2* S310F (c.929C > T) (n = 1; P083) (Table 1). *EGFR* E709A/G/K were identified in cases with another *EGFR* compound mutations, such as *EGFR* G719C/S and L858R. Conversely, two variants were covered by the AmoyDx but not covered by the ODxTT (P054 and P297) (Table 1). The ODxTT does not cover these variants, including *EGFR* L858R (c.2573_2574delinsGT) and *EGFR* exon 19 deletion (E746_T751delinsL), but these mutations were visually confirmed in raw data (Fig. 2A and B). Additionally, molecular barcode sequencing analysis was performed to verify these variants. The molecular barcode sequencing analysis yielded sufficient median molecular coverage (average: 4538; range: 1525–9678) and molecular uniformity (average: 88.2%; range: 72.6%–95.2%) (Supplementary Table 1). All these variants were detected by the molecular barcoded sequencing (Table 1). Therefore, these two misses mutations were actually present in the covered regions, but were not included in the reportable variants as these mutations are not validated by the ODxTT.

**Table 1:** Uncovered mutations by either ODxTT or AmoyDx.

ID	Gene	Variant	ODxTT	AmoyDx*	ODxTT covered variants	AmoyDx covered variants	VAF (%) (ODxTT)	Ct (AmoyDx)	MB-seq	Detail
P094	*EGFR*	G719C	Positive	Positive	Yes	Yes	20.3	24.5	Positive	–
	*EGFR*	E709A	Positive	Negative	Yes	No	20.4	NA	Positive	Not covered by AmoyDx
P320	*EGFR*	G719S	Positive	Positive	Yes	Yes	31	26.0	Positive	–
	*EGFR*	E709A	Positive	Negative	Yes	No	30.1	NA	Positive	Not covered by AmoyDx
P251	*EGFR*	G719S	Positive	Positive	Yes	Yes	28.6	26.1	Positive	–
	*EGFR*	E709G	Positive	Negative	Yes	No	25.9	NA	Positive	Not covered by AmoyDx
P269	*EGFR*	L858R	Positive	Positive	Yes	Yes	42	20.9	Positive	–
	*EGFR*	E709G	Positive	Negative	Yes	No	44	NA	Positive	Not covered by AmoyDx
P279	*EGFR*	G719S	Positive	Positive	Yes	Yes	19	29.0	Positive	–
	*EGFR*	E709K	Positive	Negative	Yes	No	19.3	NA	Positive	Not covered by AmoyDx
P083	*HER2*	S310F	Positive	Negative	Yes	No	28.3	NA	Positive	Not covered by AmoyDx
P054	*EGFR*	L858R	Negative	Positive	No	Yes	NA	25.8	Positive	c.2573_2574delinsGT is not covered by ODxTT
	*EGFR*	T790M	Positive	Positive	Yes	Yes	26.2	24.8	Positive	–
P297	*EGFR*	Exon 19 deletion	Negative	Positive	No	Yes	NA	25.6	Positive	p.E746_T751delinsL is not covered by ODxTT

*ODxTT* Oncomine Dx target test, *AmoyDx* AmoyDx pan lung cancer PCR panel, *VAF* variant allele fraction, *Ct* cycle threshold, *MB*-*seq* molecular barcode sequencing, *NA* not available.

*If AmoyDx was performed twice, the first test were used to evaluate the results.

### Discordant variant results

Since the ODxTT and the AmoyDx employ distinct detection methods, we examined the causes of six discordant variants between these two CDx tests to investigate the underlying discrepancies (Table 2). While both CDx tests cover the six variants, these variants were detected only by the AmoyDx but not detected by the ODxTT, including *EGFR* S768I (n = 1; P208)*, EGFR* exon 19 deletion (n = 1; P300), *EGFR* T790M (n = 2; P142, P240), *BRAF* V600E (n = 1; P331) and *KRAS* G12C (n = 1; P258) (Table 2). Failure of detecting *EGFR* S768I was possibly due to the presence of V774M mutation on the same allele, which may affect primer or probe binding (Fig. 2C). Notably, the raw data of the ODxTT showed no mutant alleles with coverage depth more than 1000 (Fig. 2D–H and Table 2). When retested with the AmoyDx, the Ct values for both results close to the threshold Ct value of 30 (Table 2). To further validate five mutations, we conducted NGS analysis using unique molecular barcoding on the DNA template utilized by the two CDx tests. The results showed that all five variants were confirmed as negative by molecular barcode sequencing, consistent with the data from the ODxTT. Therefore, the discordant variants observed were likely false positives in the AmoyDx.

**Table 2:** Discordant results in variants.

ID	Gene	Variant	ODxTT	AmoyDx*	ODxTT covered variants	AmoyDx covered variants	VAF (%) (ODxTT)	Ct (AmoyDx)	MB-seq	Possible reasons for discordance
P208	*EGFR*	S768I	Positive	Negative	Yes	Yes	24	NA	Positive	Nearby V774M affect primer/probe binding
P300	*EGFR*	Exon 19 deletion	Negative	Positive	Yes	Yes	0	27.4/26.3^†^	Negative	False positive by AmoyDx
P142	*EGFR*	T790M	Negative	Positive	Yes	Yes	0	29.2/28.8^†^	Negative	False positive by AmoyDx
P240	*EGFR*	T790M	Negative	Positive	Yes	Yes	0	29.7/not amplified^†^	Negative	False positive by AmoyDx
P331	*BRAF*	V600E	Negative	Positive	Yes	Yes	0	27.7/27.1^†^	Negative	False positive by AmoyDx
P258	*KRAS*	G12C	Negative	Positive	Yes	Yes	0	29.3/29.0^†^	Negative	False positive by AmoyDx

Ct threshold is set at 30 for DNA analysis (*EGFR*, *HER2*, *KRAS* and *BRAF*).

*ODxTT* Oncomine Dx target test, *AmoyDx* AmoyDx pan lung cancer PCR panel, *VAF* variant allele fraction, *Ct* cycle threshold, *MB*-*seq* molecular barcode sequencing, *NA* not available.

*If AmoyDx was performed twice, the first test were used to evaluate the results.

^†^If the results differed from those of ODxTT, AmoyDx was retested.

### Discordant fusion and exon skipping results

**Figure 3:**
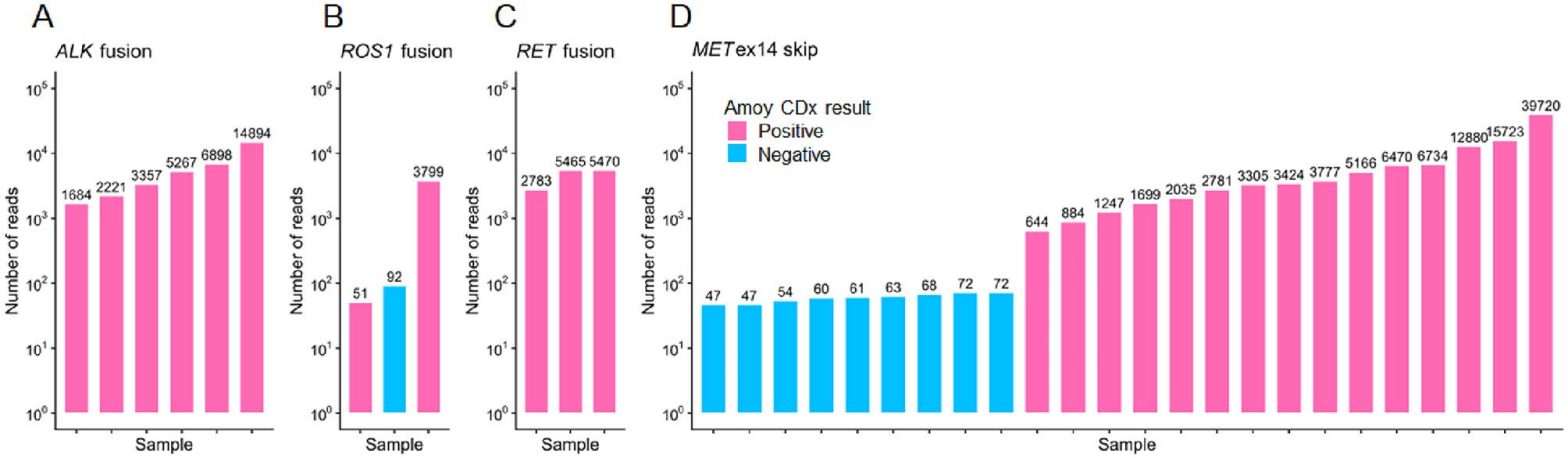
Concordance of fusion and *MET*ex14 skipping. (**A**–**D**) Bar graphs show the number of reads for *ALK* fusion (**A**), *ROS1* fusion (**B**) *RET* fusion (**C**) and *MET*ex14 skipping detected by ODxTT. Bar color indicate positive (pink) or negative (light blue) as determined by the AmoyDx. The vertical axis is on a logarithmic scale and the numbers above the bars represent the read counts.

We next investigated the discordant results of fusion and *MET*ex14 skipping. Both CDx tests detected almost all of the *ALK*, *ROS1* and *RET* fusion or *MET*ex14 skipping with high number of reads determined by the ODxTT (Fig. 3A–D). However, the AmoyDx did not detect *ROS1* fusion (n = 1) with low number of reads (Fig. 3B and D), which was the fusion of ezrin (*EZR*) exon 10 and *ROS1* exon 34 (*EZR*–*ROS1*) despite its coverage by the AmoyDx (Supplementary Fig. 3). Thus, these discrepancies were possibly attributed to the difference of the limit of detection. For *MET*ex14 skipping (n = 9), it was difficult to make a definite interpretation, but a previous study reported there were false positives associated with the low read counts observed in the ODxTT^27^.

There were nine samples detected by AmoyDx but not detected by the ODxTT (Table 3). Given the higher number of positive results for *ROS1* fusion (4.6%,6/131) and *RET* fusion (2.3% ,3/131), there was concern that these likely be false positives from AmoyDx. To this end, we retested using AmoyDx with the same RNA samples. The results showed all samples were positive in initial test, however, turned negative in second test (Table 3). Furthermore, molecular barcode sequencing did not detect these nine fusions, which was consistent with data from the ODxTT. In the initial test, Ct values were close to the cut-off value of 28 in the samples deemed positive.

**Table 3:** Discordant results in fusions.

ID	Alteration	ODxTT	AmoyDx (1st exp/2nd exp)	Ct (AmoyDx, 1st exp/2nd exp)	Well number	MB-seq
P076	*ROS1* fusion	Negative	Positive/negative	27.7/not amplified	#5	Negative
P078	*ROS1* fusion	Negative	Positive/negative	27.6/not amplified	#5	Negative
P129	*ROS1* fusion	Negative	Positive/negative	26.5/32.0	#5	Negative
P191	*ROS1* fusion	Negative	Positive/negative	26.8/31.0	#5	Negative
P331	*ROS1* fusion	Negative	Positive/negative	27.3/not amplified	#5	Negative
P335	*ROS1* fusion	Negative	Positive/negative	27.9/not amplified	#5	Negative
P094	*RET* fusion	Negative	Positive/negative	27.7/not amplified	#8	Negative
P239	*RET* fusion	Negative	Positive/negative	27.3/not amplified	#8	Negative
P268	*RET* fusion	Negative	Positive/negative	25.5/not amplified	#8	Negative

The well number indicates the corresponding location in Supplementary Fig. 4A. Ct threshold is set at 28 for RNA analysis (*ALK*, *ROS1*, *RET* and *MET*).

*ODxTT* Oncomine Dx target test, *AmoyDx* AmoyDx pan lung cancer PCR panel, *exp* experiment, *Ct* cycle threshold, *MB*-*seq* molecular barcode sequencing.

### Non-specific amplification in the AmoyDx

To explore the reasons for reproducibility issues, we examined the Ct values from the AmoyDx in all genetic alterations. The AmoyDx utilizes eight-well strips for DNA analysis and RNA analysis, targeting different genes in each well (Supplementary Fig. 4A). At the *ALK* fusion (well number #1), *EGFR* ex19 L861Q (#3), *HER2* ex20 ins (#4), *EGFR* ex20 ins (#5), *HER2* ex20 ins (#6), *ROS1* fusion (#6) and *MET*ex14 skipping (#7), there were no amplification signals in negative samples (Supplementary Fig. 4B). However, upon scrutiny of Ct values, some targets of the AmoyDx showed PCR amplification in later PCR cycles, particularly near the Ct threshold. Including the results of repeated AmoyDx tests, the negative samples with amplification signal over the Ct threshold were frequently observed in *EGFR* T790M (61.5%, 91/148), *ROS1* fusion (27.1%, 39/144), *EGFR* G719A/S/C (6.6%, 9/136) and *RET* fusion (4.4%, 6/135) (Supplementary Fig. 4B and Supplementary Table 2).

**Figure 4:**
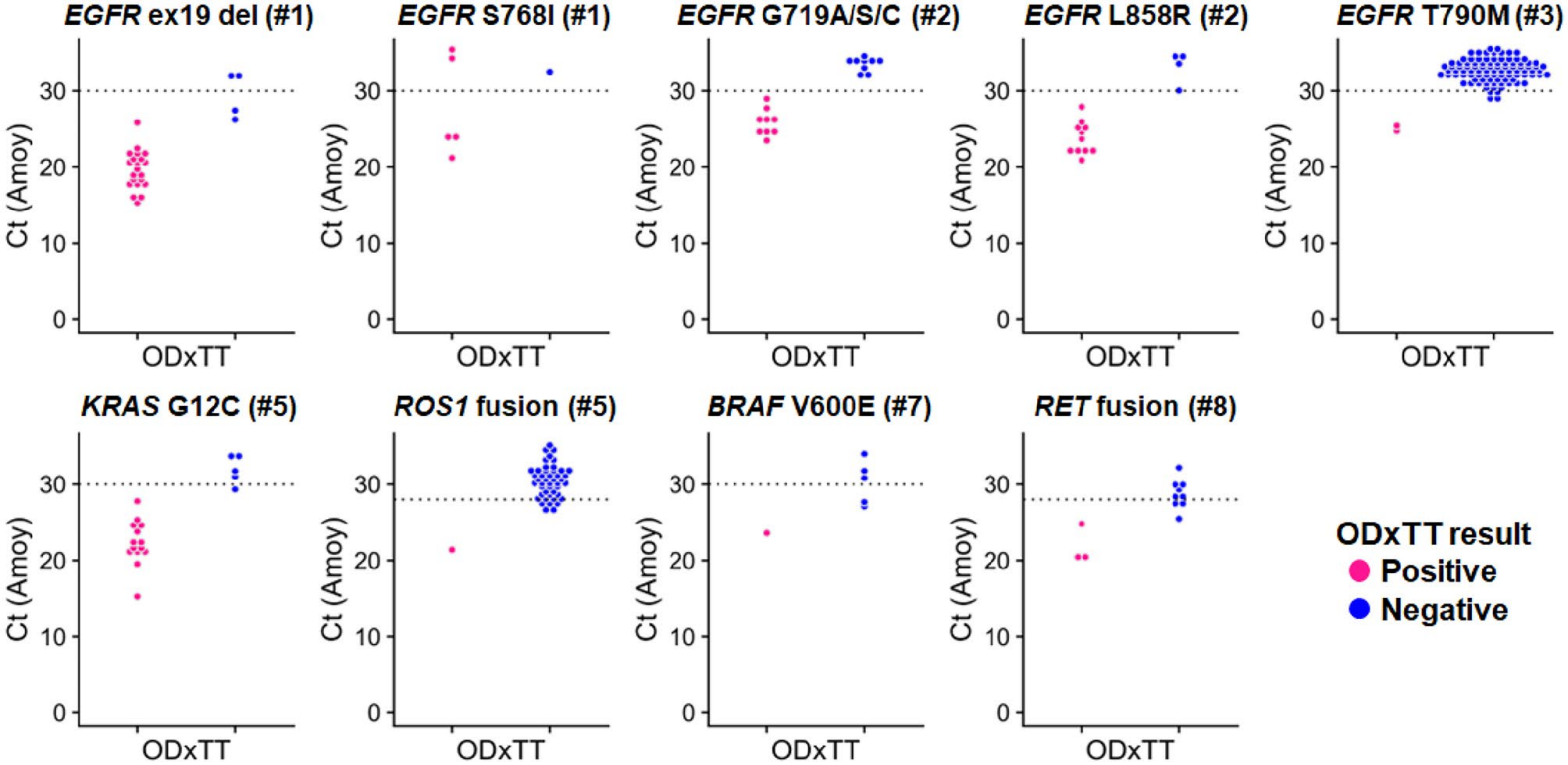
Relationship between ODxTT results and the Ct values from the AmoyDx. The dot plots indicate positive and negative samples for ODxTT and Ct values at each target measured with the AmoyDx. The subtitle number indicates the well number measured by the AmoyDx (see Supplementary Fig. 4). Colors represent positive (pink) and negative (blue) of ODxTT. Results of repeated the AmoyDx tests are included in this graph. The dotted lines indicate the Ct threshold, set at 30 for DNA analysis and 28 for RNA analysis.

To distinguish accurate results, we examined the distribution of the Amoy Ct values using the ODxTT results as reference data. Samples that were positive on the ODxTT tended to have low Ct values on AmoyDx, while Samples that were negative on the ODxTT tended to have high Ct values on AmoyDx (Fig. 4). However, some samples were close to the Ct threshold in *EGFR* exon19 deletion, *KRAS* G12C and *RET* fusion, making it difficult to make a clear distinction (Fig. 4). To analyze samples expected to be negative for known mutations, we tested normal lung tissues from 20 patients with pneumothorax using the AmoyDx and the ODxTT. With the AmoyDx, two out of the 20 samples were positive (*KRAS* G12C [n = 1] and *ROS1* fusion [n = 1]) (Supplemental Fig. 5). The Ct values for these positive samples were near the threshold line. Additionally, although these Ct values were below cut-off value, amplification signals were detected in several other samples (Supplemental Fig. 5). In contrast, all 20 samples tested negative when evaluated by the ODxTT assay. These findings suggest a possible risk of false positives due to non-specific reactions during the PCR process at some targets in the AmoyDx.

## Discussion

In this study, we conducted direct performance comparison between the ODxTT and the AmoyDx to evaluate the concordance. The results showed that the overall positive concordance rate between the ODxTT and the AmoyDx was 89.0% (89/100), and the negative concordance rate was 98.5% (937/951), with 97.6% (1026/1051) overall concordance rate. We also showed a correlation between the VAF in the ODxTT and the Ct values in the AmoyDx. However, we identified 2.4% (25/1051) of discordant results between two CDx tests and a potential for false positives in AmoyDx, which are challenging issues for the future. Due to the cost implications for hospitals, simultaneous testing with the ODxTT and the AmoyDx is not currently performed given the current insurance system. In the context that the choice of one of the two tests is unavoidable, our insight into the substantial differences between the two CDx tests provides clinical benefits for lung cancer patients. To our knowledge, this study could represent the first report of two representative CDx tests simultaneously, shedding light on the pitfalls of these tests. This finding contributes to a better understanding of test characteristics, leading to a redefinition of result interpretation and increased awareness. It also underscores the contribution to the further development of improved molecular diagnostics for lung cancer.

The problem of the ODxTT is that certain mutations are not included in the reporting program because only validated hotspots are considered reportable variants^28,29^. Our simulation analysis showed that *EGFR* rare mutations were not detected by the ODxTT^26^. Consistent with this, we identified dinucleotide variants in *EGFR* L858R and rare exon 19 deletion. These rare mutations are not included in the ODxTT detectable mutation list, causing a miss in the report. Although the missing result could be detected by visual confirmation in the raw data, it requires additional efforts. Nevertheless, in order to treat lung cancer patients with effective drugs, confirmation of raw data should be necessary. For pan-driver-negative lung cancer cases in particular, we recommend manual review of the raw data from the ODxTT. Hopefully, some rare mutations will be included in the reported mutation list, and the ODxTT can detect more rare mutations in the future. Another issue is that samples positive for *MET*ex14 skipping with a low number of reads were likely to yield false positive results. This issue has been previously highlighted^27^. One proposed explanation is that low basal rates of alternative splicing in *MET* amplified/ overexpressed tumor, potentially due to low-level splicing errors, can cause these misleading results^30^. In this context, a higher number of *MET*ex14 skipping reads needs to be set as a threshold for detecting true skipping events. In our analysis, *MET*ex14 skipping reads exceeding 644 were consistent with the AmoyDx results. To date, *MET*ex14 skipping results from the ODxTT are not approved for companion diagnostic labeling in Japan. For future companion diagnostic approval of MET inhibitors using the ODxTT, it will be necessary to establish suitable cutoff values.

Our results showed that the AmoyDx has the potential for false positive results. Previous study indicated that false positives were attributed to the artificial substitutions during formalin fixation^31^, but this possibility was not consistent with the finding that no mutant allele was present in the raw sequencing data of the the ODxTT. Another possibility is related to the ARMS method, which uses a 3' mismatch base of the mutant allele. There is a potential for non-specific reactions due to misannealing at this site^32–34^. In the AmoyDx, the PCR is performed for 10 cycles at 2nd stage and 36 cycles at 3rd stage, for a total of 46 cycles of PCR reaction. It means that the actual number of PCR cycles corresponds to the Ct value measured by AmoyDx plus 10 cycles, indicating there is a possibility of non-specific amplification during the PCR. In fact, the reproducibility of some results could not be confirmed in repeated AmoyDx tests (Table 2). Notably, in 20 normal lung tissues form pneumothorax patients, the AmoyDx detected *KRAS* G12C mutation (n = 1) and *ROS1* fusion (n = 1). Therefore, if the Ct value is near the cut-off value, it may be necessary to check the reproducibility of the AmoyDx (Fig. 4). However, increasing cost of repeated test is a real concern. If a sample is truly positive but has a low tumor content and a low quantity of extracted nucleic acid, the Ct value may be near the threshold line. In any case, caution is exercised when interpreting the AmoyDx results.

Another concern is the discrepancy where samples tested twice by the AmoyDx were positive results but negative by the ODxTT. To examine these issues, molecular barcoding sequencing was performed and revealed that the AmoyDx data may be false positives. Since the primer sequence information is not publicly available, the actual reason for this difference remains unclear. In almost all hospitals, if the AmoyDx is outsourced and determined in a one-time test, there would be a remaining concern of a false-positive result. In this context, there is a risk of incorrectly administering expensive treatments that provide less benefit to the patient, posing challenges in terms of healthcare resources, costs, and patient burden.

This study has several limitations. First, due to the limited number of positive specimens in certain target regions, it was not possible to examine the positive concordance rate with a large number of specimens. Second, the data for this study are from a single institution. Future studies with data from multi-center study may provide a more comprehensive and consistent understanding. Third, some discordant data lacked a clear explanation for the discrepancies. Fourth, as repeated detection of similar Ct values in retesting with the AmoyDx, it still remained the possibility of sample contamination or detection of a low-level variant background by the AmoyDx. We performed molecular barcode-based verification, and given that AmoyDx (ARMS-PCR) has a limit of detection of 1–5% while the molecular barcode method has a limit of detection of 0.5%, these possibilities are considered low but cannot be completely ruled out.

In summary, our comparative analysis sheds light on the strengths and limitations of the ODxTT and the AmoyDx in lung cancer molecular profiling. To ensure quality improvement and reliability in molecular diagnostics, it is imperative to continuously monitor and evaluate new versions or updates of these CDx tests. By proactively addressing the challenges identified and pursuing improvements, the field can progress toward more effective and reliable CDx tests. These efforts hold the promise of ultimately benefiting lung cancer patients and guiding the development of precision medicine.

## Method

### Patients and samples

We have tested 333 samples from 325 lung cancer patients using the ODxTT (Thermo Fisher Scientific, Waltham, MA, USA) from December 2019 to December 2023. After tested using the ODxTT, extracted DNA and RNA samples were stored at − 80 °C until subsequent analysis. Of these frozen DNA and RNA, we randomly selected 131 samples from 131 lung cancer patients and analyzed using the AmoyDx. The characteristics of the patients were as follows: median age of 73 (interquartile range [IQR]: 65–78), 53 women (40%) and 78 men (60%), tumor stages were categorized into stage I (n = 14, 11%), II (n = 13, 10%), III (n = 26, 20%), IV (n = 76, 58%), and unknown (n = 2, 2%), histological types including adenocarcinoma (n = 101, 77%), squamous cell carcinoma (n = 13, 10%), carcinoid (n = 1, 0.8%), malignant tumor (n = 1, 0.8%), neuroendocrine tumor (n = 1, 0.8%), NSCLC (n = 13, 10%), NSCLC not otherwise specified (n = 1, 0.8%). The sample types comprised formalin-fixed, paraffin-embedded (FFPE) tissues (n = 124, 95%), cytology specimens (n = 5, 4%) and cell blocks (n = 2, 2%).

### ODxTT

The ODxTT was performed in our hospital at the division of genetics and clinical laboratory according to the manufacturer’s instructions with minor modification^19^. Briefly, two 5-μm thick sections were prepared for specimens obtained by surgical resection, and twelve 5-μm thick sections were prepared for specimens obtained by other methods. If the area of tumor cells were small surrounding the adjusted non-tumor area, we macrodissected tumor area on some FFPE tissues.

DNA and RNA were extracted using the ion Torrent Dx total nucleic acid isolation kit from FFPE tissues, cytology specimens and cell block^19^. All samples were treated with protease and the lysates were filtered through a filter cartridge. The filter cartridges were then washed with wash buffer, and RNA and DNA were extracted. RNA samples were treated with DNase prior to extraction. DNA and RNA concentrations were measured on Qubit 3.0 with the Ion Torrent Dx DNA Quantification Kit or the Ion Torrent Dx RNA quantification kit (Thermo Fisher Scientific).

DNA concentration was diluted to 0.83 ng/µL and RNA concentration was diluted to 1.43 ng/µL with a dilution solution. A total of 10 ng of DNA and RNA was used for library preparation. If the extracted DNA or RNA concentration was less than the recommended concentration, we used the extracted samples directly with input of less than 10 ng. Complementary DNA (cDNA) was synthesized using the Ion Torrent Dx cDNA Synthesis Kit. Target regions were amplified with a DNA panel and a RNA panel in the Ion PGM Dx Library Kit by multiplex polymerase chain reaction (PCR)^20^. Amplified amplicons were partially digested with FuPa reagent and then ligated with barcodes to each sample. The library was purified and equalized with the Ion PGM Dx Library Equalizer Kit. Emulsion PCR and library enrichment were performed with the Ion OneTouch Dx Template Kit on the Ion OneTouch Dx instrument and the Ion OneTouch ES Dx instrument. Sequencing was conducted using the Ion PGM Dx sequencer (Thermo Fisher Scientific) with the Ion PGM Dx Sequencing Kit and the Ion PGM 318 Dx Chip. Data analysis was conducted with the Torrent Suite Dx software with an assay definition file. The data of all samples were passed to the control samples (DNA control, RNA control and No template control, Control Fragment-1) of sequencing quality. VAF, the number of fusion reads and skipping reads were searched from reports on the Torrent Suite Dx. Some variants were visually confirmed with the integrative genomics viewer (IGV)^35^.

### AmoyDx

We conducted using the AmoyDx pan lung cancer PCR panel (Amoy Diagnostics Co., Ltd., Xiamen, China) according to the manufacture’s instruction. Nucleic acid concentration was measured using the NanoDrop spectrophotometer (Thermo Fisher Scientific). We diluted RNA concentration to 10 ng/µL and DNA to 2 ng/µL with nuclease free water (Thermo Fisher Scientific). RNA was reverse transcribed into cDNA at 42 °C for 1 h and 95 °C for 5 min using allele-specific primers. Real-time PCR analysis was conducted on the QuantStudio 7 (Thermo Fisher Scientific). The PCR conditions were set as follows: 1st stage: 42 °C for 5 min, 94 °C for 5 min for one cycle; 2nd stage: 95 °C for 25 s, 64 °C for 20 s, 72 °C for 20 s for 10 cycles; 3rd stage: 93 °C for 25 s, 60 °C for 35 s, 72 °C for 20 s for 36 cycles. Fluorescence intensities were measured during the step 3rd stage, and the threshold line was set at 5% of the fluorescent maximum value at the 36th cycle of the positive control. Subsequently, Ct value of each well was determined and the test result was judged as following criteria: For RNA analysis, Ct < 28 was considered positive, while Ct ≥ 28 was considered negative. For DNA analysis, Ct < 30 was considered positive, Ct ≥ 33 was considered negative, and in case 30 ≤ Ct < 33, the delta Ct between the sample and external control was calculated for determination. AmoyDx applies multiplex PCR to measure multiple target regions in the same well, it cannot discriminate the nucleotide change and fusion partner in some genes. In the package insert, the limit of detection is 1–5% VAF for variants and 150 copies per reaction for fusion and *MET*ex14 skipping.

### NGS with a unique molecular barcode

Samples with discordant results were validated with molecular barcode sequencing analysis. DNA was treated with uracil-DNA glycosylase in advance. The library preparation and sequencing were conducted on the Ion Torrent Genexus System in accordance with the manufacturer's instructions (Thermo Fisher Scientific). We used Oncomine precision assay GX (Thermo Fisher Scientific) or in-house primer pools for lung cancer or as previously described^36^. DNA and RNA concentrations were diluted to 0.67 ng/µL in nuclease-free water and 40µL of samples were used for analysis. The Genexus Library Strips 1 and 2-HD (Thermo Fisher Scientific) and Genexus Templating Strips 3-GX5™ and 4 (Thermo Fisher Scientific) were incubated at room temperature for 30 min before being loaded into the sequencer. The Genexus Barcodes 1–32 HD was also set in the sequencer.

Raw signal data from the sequencing analysis were processed using the standard pipeline (Torrent Suite version 6.8.1.1) in the Torrent Suite Software running on the Genexus Software (Thermo Fisher Scientific). The data processing pipeline, read alignment, mapping to genome, and plugin analysis (coverage analysis and molecular coverage analysis) were executed. Variant analysis was performed with the following parameter in “Variants” tab: (i) Call include “PRESENT” (ii) nonsynonymous variant or fusion (iii) filter gene of interest. Subsequently, discordant results were search for identifier of the Catalogue of Somatic Mutations in Cancer and checked the variants in each sample.

### Analysis of normal lung tissue

We analyzed 20 patients with pneumothorax (18 men, 2 women), with a median age of 69 years (IQR: 61–74). Lung tissue resected during pneumothorax surgery was formalin-fixed and paraffin-embedded for histological analysis. Pathological examination confirmed the absence of malignant findings in the lung tissue. DNA and RNA were extracted from these FFPE samples using the MagMAX™ FFPE DNA/RNA Ultra Kit (Thermo Fisher Scientific) on the KingFisher Duo Prime (Thermo Fisher Scientific) according to the manufacturer's instructions. Nucleic acid concentrations were measured using Qubit and NanoDrop, as described above. The nucleic acids were diluted to the required concentrations and analyzed using the ODxTT or the AmoyDx platforms.

### Ethics statement

The Institutional Review Board of the Clinical Research and Genome Research Committee of Yamanashi Central Hospital approved this retrospective study (Approval No. G-2018-4). Informed consent was obtained from all subjects or their legal guardian to participate in the study. All study procedures were performed in accordance with the relevant guidelines and regulations, and as set out in the Helsinki Declaration.

### Data processing, visualization and statistical analysis

IQR calculations, statistical analyses, and correlation analyses were performed using R version 4.1.1 (http://www.r-project.org/). Statistical significance was defined as a p value < 0.05. The following R packages were used for data cleaning, analysis, and visualization: ggplot2 (v3.4.4), tidyverse (v2.0.0), ggpubr (v0.6.0), stringr (1.5.0), patchwork (v1.1.1), cowplot (v1.1.1), gtsummary (v1.5.2) and epiR (v2.0.66).

### Supplementary Information

Supplementary Information.

## Data Availability

The source data underlying figures and tables are available upon reasonable request from the corresponding author.
