# Supplementary material for: Comparison of diagnostic performance between Oncomine Dx target test and AmoyDx panel for detecting actionable mutations in lung cancer
Source: Sci Rep. 2024 May 30;14:12480. doi: 10.1038/s41598-024-62857-8 (PMC11139982; doi:10.1038/s41598-024-62857-8)
Supplement: Supplementary file 1 — Supplementary Information. [file 41598_2024_62857_MOESM1_ESM.pdf]

Supplementary information

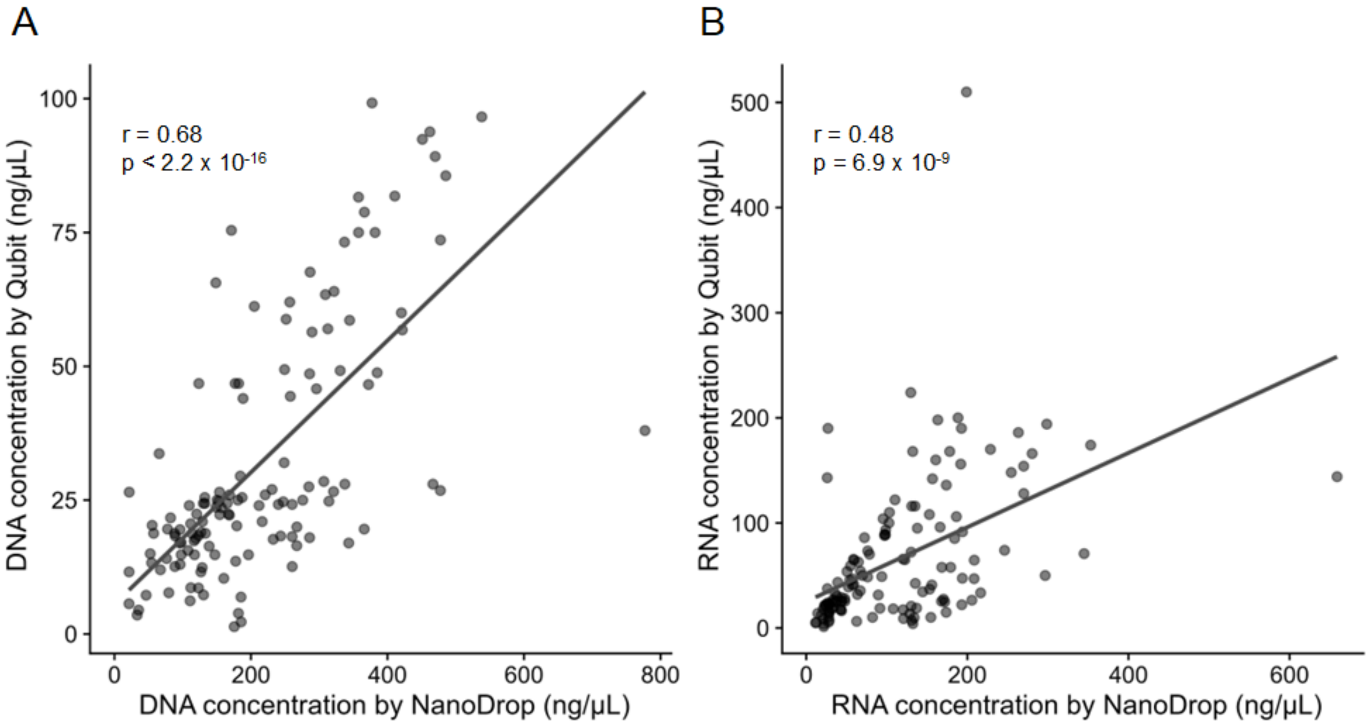

Supplementary Figure 1. Correlation of nucleic acid concentrations

(A, B) Scatter plot shows the correlation between nucleic acid concentrations (ng/μL) measured by NanoDrop and Qubit. Positive correlations were observed for both DNA (A) and RNA (B) concentrations measured by two assays. r, correlation coefficient.

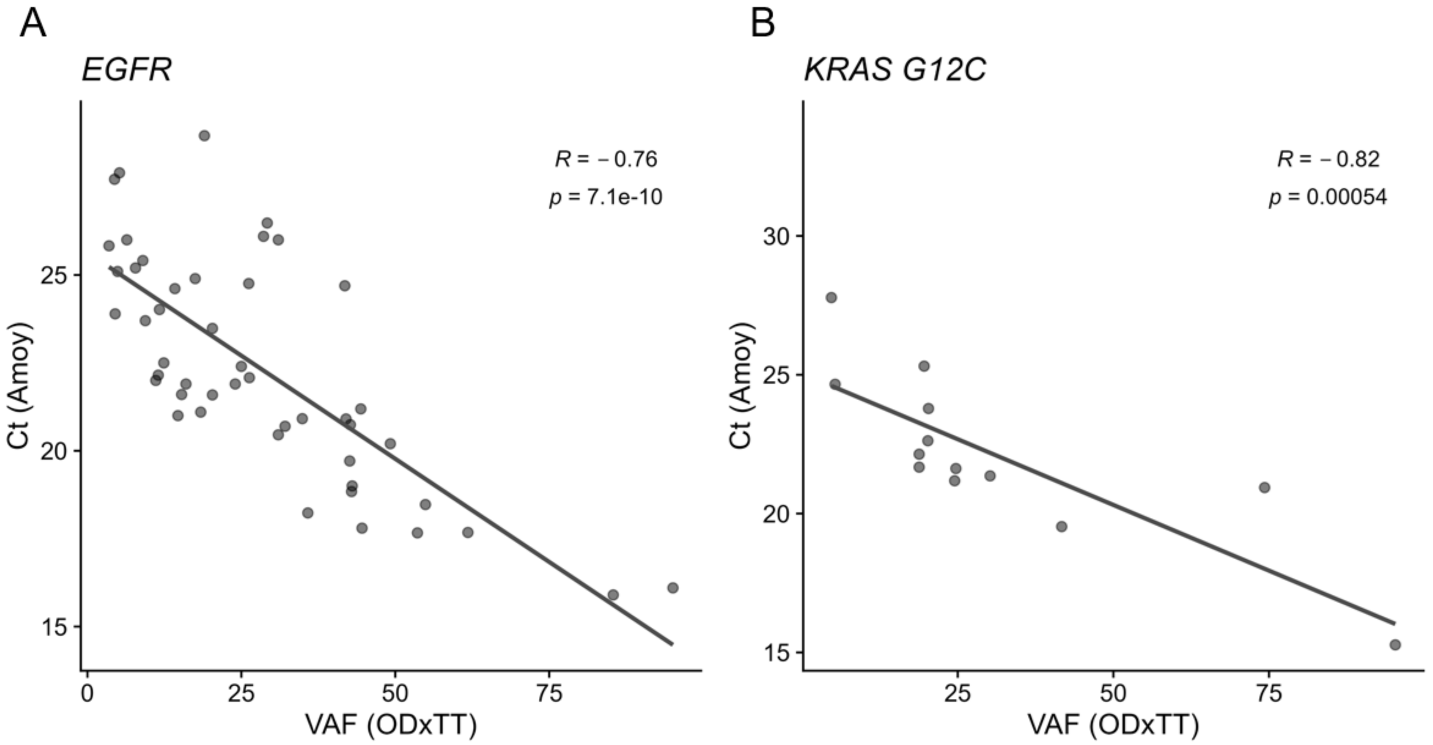

Supplementary Figure 2. Correlation between VAF and Ct values

(A, B) Scatter plot shows the correlation between variant allele fraction (VAF) measured by ODxTT and threshold cycle (Ct) values measured by AmoyDx. Results are shown for *EGFR* (n=47, A) and *KRAS* G12C (n=13, B), two genes with a high number of mutations detected by both CDx tests. Positive correlations were observed between VAF and Ct values. R, correlation coefficient.

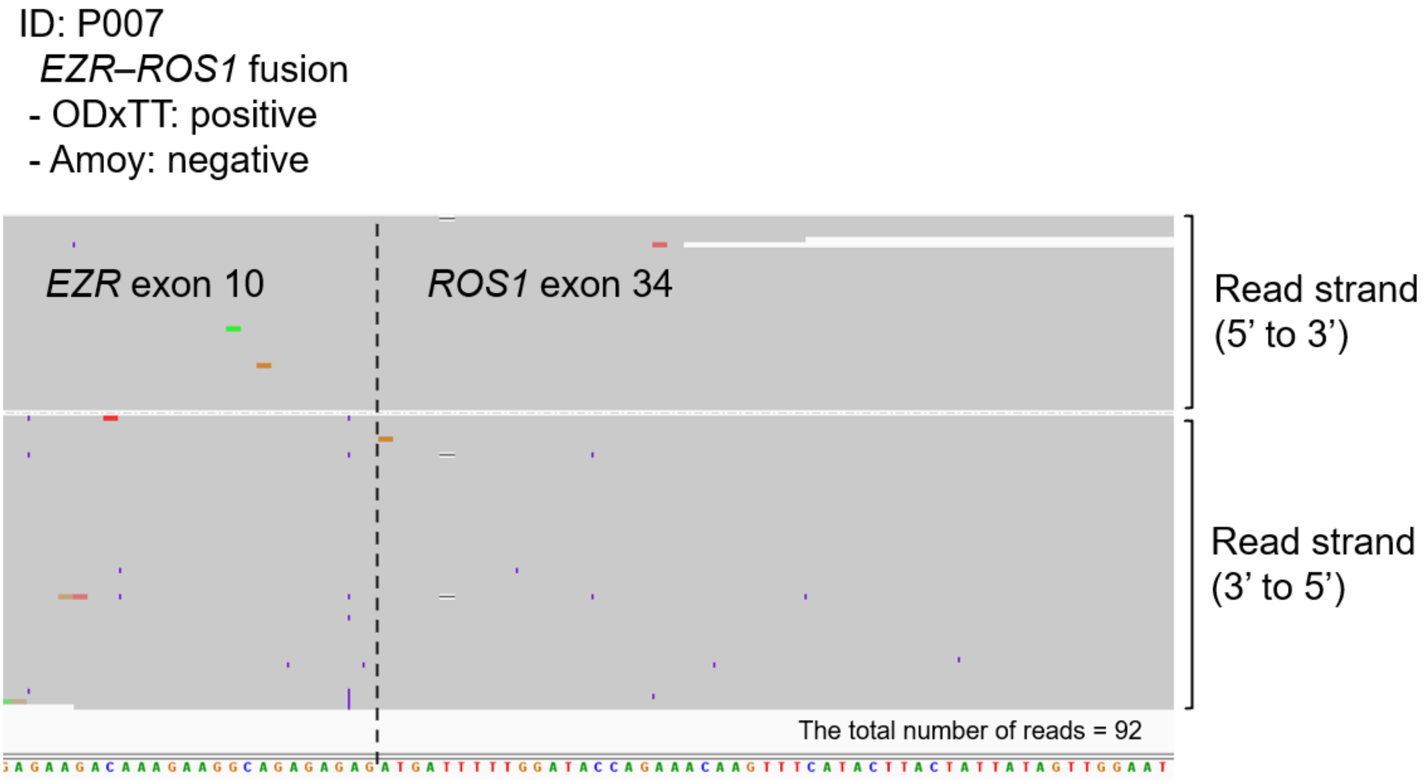

Supplementary Figure 3. Visual confirmation of *EZR–ROS1* fusion

The read alignment data is presented using the Integrative Genomics Viewer. The dashed lines indicate the boundary between *EZR* exon 10 and *ROS1* exon 34. This fusion was identified with 92 reads, showing positive in ODxTT and negative in AmoyDx.

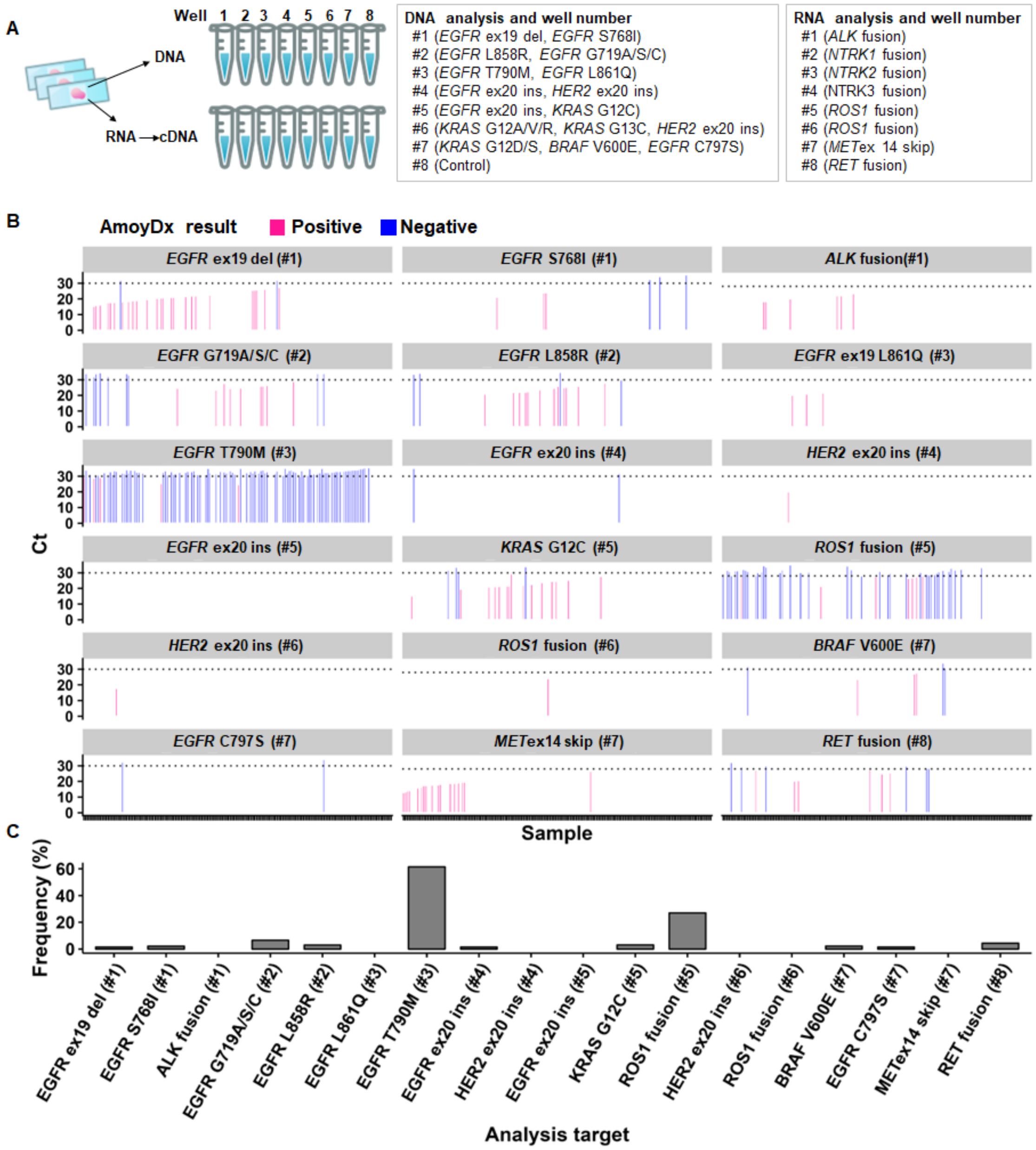

Supplementary Figure 4. Ct values in lung tumor tissues evaluated by the AmoyDx

(A) The scheme shows the multiplex PCR reaction by AmoyDx. DNA and RNA analysis is performed using an eight-well strip, with each well corresponding to the detection of specific genetic alterations. **(B)** Bar graph indicates the Ct values detected by AmoyDx for each well. Bar colors represent positive (pink) and negative (blue). Results of repeated AmoyDx tests are included in this graph. The dotted lines indicate the Ct threshold, set at 30 for DNA analysis and 28 for RNA analysis. Sample with Ct value of zero indicated either

sample with no amplification or that the sample did not reach the threshold line. (**C**) The frequency of negative samples with amplification signal over the Ct threshold.

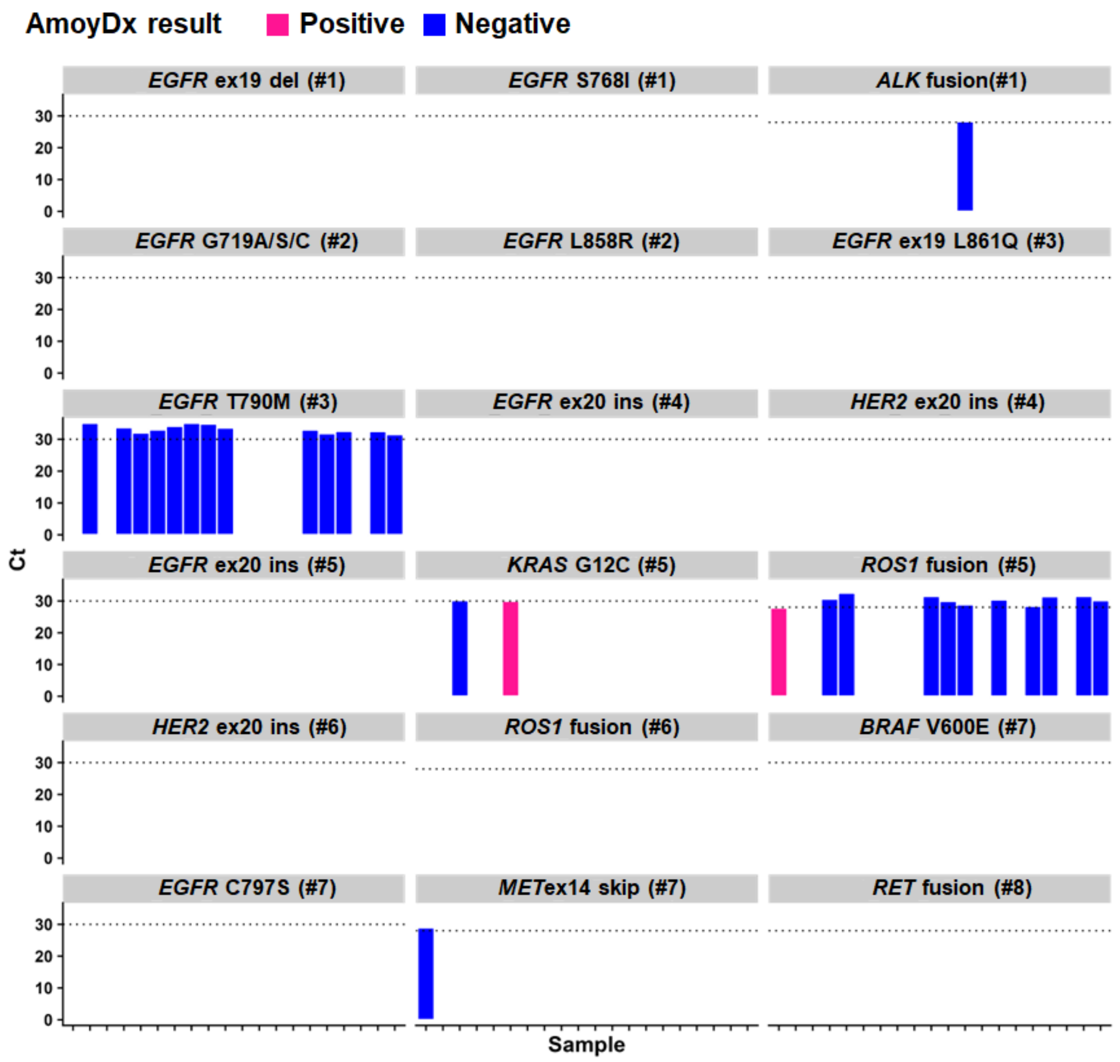

Supplementary Figure 5. Ct values in normal lung tissues evaluated by AmoyDx

Normal lung tissue samples obtained from 20 patients with spontaneous pneumothorax were analyzed using AmoyDx. Bar graph indicates the Ct values detected by AmoyDx for each well. Bar colors represent positive (pink) and negative (blue). The dotted lines indicate the Ct threshold, set at 30 for DNA analysis and 28 for RNA analysis. Sample with Ct value of zero indicated either sample with no amplification or that the sample did not reach the threshold line.

Supplementary Table 1. Coverage analysis of molecular barcode sequencing

ID	Mapped reads	On target	Coverage depth	Uniformity	Median molecular coverage	Molecular uniformity	Median reads per functional molecule	Median perc functional reads
P054	4,628,641	98.1%	82,890	96.4%	2,654	90.3%	21.3	88.8%
P083	4,006,875	97.7%	68,338	95.7%	1,525	88.7%	27.8	89.6%
P094	4,226,254	98.5%	74,432	95.3%	1,859	91.9%	25.6	89.8%
P142	3,244,278	98.9%	57,868	88.4%	4,522	72.6%	4.4	52.1%
P208	3,073,991	98.4%	52,650	95.3%	5,160	79.0%	12.2	73.1%
P240	3,135,334	98.2%	53,308	93.4%	5,084	82.3%	11.3	72.5%
P251	4,734,740	97.8%	83,905	96.3%	3,976	91.9%	13.6	88.5%
P258	3,350,697	98.2%	57,733	92.9%	6,836	93.6%	8.1	69.9%
P269	4,346,854	97.8%	76,374	95.6%	4,859	93.6%	10	87.5%
P279	4,272,183	98.3%	75,560	95.3%	2,904	93.6%	17.6	88.6%
P297	4,312,081	97.7%	75,937	96.3%	2,998	95.2%	17.5	88.9%
P300	3,023,128	98.5%	51,520	93.4%	6,602	85.5%	7.9	70.8%
P320	4,913,228	97.4%	86,233	96.4%	4,870	95.2%	11.1	87.7%
P331	3,193,736	98.2%	55,206	93.8%	9,678	80.7%	4.3	50.2%

Supplementary Table 2. Frequency of the samples above Ct threshold

Analysis target	Total number of analyzed samples	Number of samples above Ct threshold	Frequency (%)
EGFR ex19 del (#1)	137	2	1.5
EGFR S768I (#1)	134	3	2.2
ALK fusion (#1)	130	0	0
EGFR G719A/S/C (#2)	136	9	6.6
EGFR L858R (#2)	135	4	3
EGFR L861Q (#3)	133	0	0
EGFR T790M (#3)	148	91	61.5
EGFR ex20 ins (#4)	135	2	1.5
HER2 ex20 ins (#4)	131	0	0
EGFR ex20 ins (#5)	133	0	0
KRAS G12C (#5)	131	4	3.1
ROS1 fusion (#5)	144	39	27.1
HER2 ex20 ins (#6)	130	0	0
ROS1 fusion (#6)	130	0	0
BRAF V600E (#7)	131	3	2.3
EGFR C797S (#7)	133	2	1.5
*MET*ex14 skip (#7)	132	0	0
RET fusion (#8)	135	6	4.4

Note: Ct threshold is set at 30 for DNA analysis (*EGFR*, *HER2*, *KRAS* and *BRAF*) and 28 for RNA analysis (*ALK*, *ROS1*, *RET* and *MET*).
